# Raster Orientation Effects on the Adhesion of iCVD-Deposited PSA Thin Films on FDM-Printed PLA

**DOI:** 10.3390/polym18030371

**Published:** 2026-01-30

**Authors:** Aydın Güneş, Kurtuluş Yılmaz, Mehmet Gürsoy, Mustafa Karaman

**Affiliations:** 1 Department of Mechanical Engineering, Abdullah Gül University, Kayseri 38080, Türkiye; aydingns@hotmail.com; 2 Department of Chemical Engineering, Konya Technical University, Konya 42030, Türkiye; kurtulusyilmaz3@gmail.com (K.Y.); mkaraman@ktun.edu.tr (M.K.); 3 Nanotechnology and Advanced Materials Development Application and Research Center, Konya Technical University, Konya 42030, Türkiye

**Keywords:** iCVD, polymeric thin film, pressure-sensitive adhesive, coating, raster orientation, adhesion performance

## Abstract

The adhesion performance of pressure-sensitive adhesive (PSA) thin films on additively manufactured polymers is strongly governed by surface anisotropy induced during printing. In this study, PSA thin films based on 2-ethylhexyl acrylate (EHA) and acrylic acid (AA) were deposited by initiated chemical vapor deposition (iCVD) onto fused deposition modeling (FDM) printed PLA substrates with different raster orientations (0°, 30°, 60°, and 90°). The deposited films exhibited high optical transparency on glass, and thicknesses consistent with the targeted deposition. Adhesion performance was evaluated using tensile and three-point bending tests, revealing a strong dependence on raster orientation. The 0° raster orientation yielded the highest shear adhesion strengths, reaching 12.03 N/cm^2^ under tensile loading and 4.59 N/cm^2^ under bending, along with the largest failure displacements. In contrast, specimens printed at 90° exhibited an approximately 47% reduction in tensile shear adhesion strength and limited deformation prior to failure. SEM analysis showed that raster alignment parallel to the loading direction promoted extensive adhesive deformation and PSA fibrillation, whereas higher raster angles resulted in predominantly interfacial debonding. These results demonstrate that raster orientation is a critical design parameter for tuning PSA adhesion on FDM-printed PLA substrates without modifying adhesive chemistry.

## 1. Introduction

Additive manufacturing (AM) enables the fabrication of three-dimensional components with complex geometries through layer-by-layer deposition using computer-aided design and 3D printing technologies. Among various AM techniques, fused deposition modeling (FDM) has become widely adopted due to its accessibility, dimensional accuracy, rapid prototyping capability, and broad range of printable polymeric materials. As a result, extensive research has focused on the mechanical characterization of FDM-printed polymers and on the influence of processing parameters such as printing speed, build and raster orientations, and layer thickness on bulk mechanical performance [1]. Beyond bulk properties, the surface characteristics of FDM-printed polymers are increasingly recognized as critical factors in applications involving joining, coating, and functional surface integration. Adhesive bonding, in particular, has been widely investigated as an effective method for assembling additively manufactured components and hybrid structures. Accordingly, numerous studies have examined the mechanical behavior of adhesively bonded joints using configurations such as single-lap joints, emphasizing the effects of bond-line thickness, overlap length, surface morphology, and loading conditions on joint performance [2,3,4,5,6,7].

In the context of additively manufactured substrates, several researchers have demonstrated that printing parameters and surface morphology significantly influence adhesive performance. Kovan et al. [8] reported that layer thickness and print orientation strongly affect the bonding strength of adhesively bonded FDM components. Garcia and Prabhakar [9] showed that additively manufactured surface textures can be used to tailor interfacial behavior and improve shear strength, while Spaggiari and Denti [10] highlighted the dominant role of surface-induced stress concentrations in governing failure mechanisms. These studies collectively indicate that the surface architecture imposed by the FDM process plays a decisive role in adhesion-related performance. However, the majority of existing studies have focused on structural adhesives and macroscopic joint strength, where failure is governed by bulk adhesive properties and joint mechanics. In contrast, pressure-sensitive adhesives (PSAs) operate through fundamentally different mechanisms, relying on intimate interfacial contact, viscoelastic deformation, and energy dissipation at the surface [11]. Consequently, PSA performance is particularly sensitive to surface topography and directional features, such as the raster-induced anisotropy inherent to FDM-printed surfaces. Despite this sensitivity, the influence of raster orientation on PSA-type thin-film adhesion has received limited attention. While raster-orientation effects have been studied for structural adhesives and bulk joints, systematic investigations focusing on PSA thin-film adhesion remain scarce.

Conventional approaches for depositing thin adhesive layers on polymer substrates typically include solvent-based coating methods such as spin coating, spray coating, dip coating, or film lamination. While these techniques are widely used, they often suffer from limited thickness control, solvent-related defects, and non-uniform coverage, particularly on rough or anisotropic surfaces. These limitations become more pronounced for additively manufactured substrates, where raster-induced topography can lead to uneven infiltration, pore filling, or local thickness variations, thereby obscuring surface-driven adhesion effects, as described in the vapor-phase polymer coating literature [12,13,14,15]. Consequently, conventional deposition methods are not well suited for systematically isolating the influence of surface morphology on PSA-type thin-film adhesion.

Surface modification techniques, including chemical treatments and plasma activation, have been explored to enhance adhesion on additively manufactured polymers [16]. While effective, these approaches often alter surface chemistry and can obscure the intrinsic contribution of surface morphology. Initiated chemical vapor deposition (iCVD), on the other hand, enables the deposition of conformal, solvent-free, ultrathin polymer films while preserving the underlying surface microstructure [17,18,19,20]. This makes iCVD particularly suitable for investigating morphology-driven adhesion effects without introducing additional chemical complexity.

In PSA design, acrylate-based systems are among the most widely used due to their balanced viscoelastic response, chemical stability, and tunable adhesion characteristics. In particular, polymers based on 2-ethylhexyl acrylate (EHA) provide low glass transition temperatures and high chain mobility, while the incorporation of acrylic acid (AA) introduces polar functional groups that enhance interfacial interactions and cohesive strength [21,22,23,24,25]. Such P(EHA-co-AA) therefore offers a well-established and representative platform for investigating surface-structure-driven adhesion effects.

In this study, iCVD-deposited PSA thin films based on EHA and AA were applied onto fused deposition modeling (FDM)-printed polylactic acid (PLA) substrates with different raster orientations fabricated under identical printing conditions. The PSA coatings were deposited with uniform chemistry and thickness in order to isolate the effect of raster-induced surface anisotropy. In contrast to approaches that emphasize bulk joint strength, the present work focuses on surface-controlled adhesion mechanisms. By examining PSA thin films, whose performance is governed by interfacial contact, viscoelastic deformation, and fibrillation, this study demonstrates how raster orientation directly influences adhesion behavior. Such effects are particularly relevant in applications where PSAs function as thin interfacial layers, and adhesion is determined by surface contact quality rather than overall joint dimensions. Examples include additively manufactured fixtures, modular components, and functional assemblies where surface morphology is dictated by printing parameters.

## 2. Materials and Methods

### 2.1. Fabrication of FDM-Printed PLA Substrates

Polylactic acid (PLA) substrates were fabricated using a FDM process to investigate the effect of raster orientation on adhesion behavior. All specimens were printed using a Flash Forge AD5X 3D printer equipped with a 1.75 mm PLA filament. No post-processing or surface treatment was applied in order to preserve the intrinsic surface morphology generated during printing. Rectangular PLA substrates were designed using SolidWorks software (https://www.solidworks.com, 10 August 2025) and exported as STL files for slicing. The slicing process was carried out using Orca Slicer, which generated the G-code for printing. To ensure consistent material deposition and surface quality across all samples, the printing parameters were kept constant, including a layer thickness of 0.2 mm, an infill density of 80%, a layer width of 0.4 mm, a print speed of 200 mm s^−1^, a nozzle temperature of 210 °C, and a build plate temperature of 60 °C.

**Figure 1:**
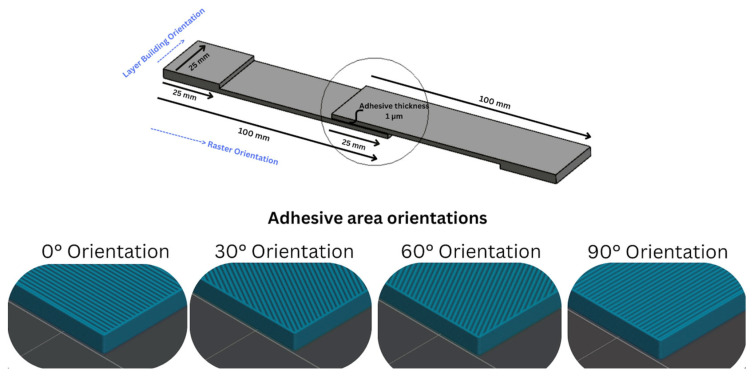
Schematic illustration of the test geometry and raster orientations (0°, 30°, 60°, and 90°) of FDM-printed PLA substrates used for tensile and three-point bending adhesion tests.

Raster orientation was selected as the primary variable in this study. The PLA substrates were printed with raster angles of 0°, 30°, 60°, and 90°, representing commonly employed deposition orientations in FDM processing. These orientations were chosen to generate distinct, directionally anisotropic surface morphologies while maintaining identical bulk material properties. In addition, tensile tests of uncoated FDM-printed PLA used in adhesion tests were performed according to ASTM-D638 [26] with rate of 1 mm/min (Shimadzu, Kyoto, Japan/Universal), and the effect of different raster orientations was determined before the adhesion tests. A schematic representation of the raster orientations used is shown in Figure 1.

### 2.2. Deposition of PSA Thin Films by iCVD

**Figure 2:**
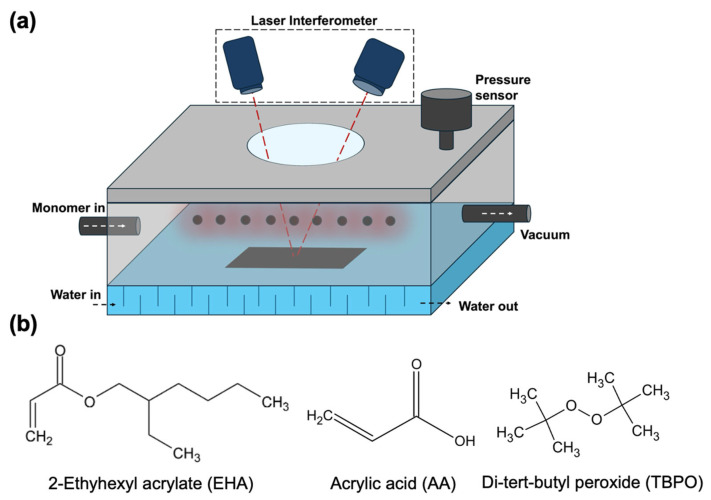
(**a**) The schematic representation of the iCVD system, (**b**) the chemical structure of EHA, AA and TBPO.

PSA thin films were deposited using an iCVD reactor supplied by Nanofors (Konya, Türkiye), featuring a hydraulically actuated top-loading lid for substrate placement. The reactor chamber had internal dimensions of 70 × 55 × 15 cm^3^. The schematic representation of the iCVD is presented in Figure 2a. The reactor was evacuated to a base pressure of 5–10 mTorr using an oil-sealed rotary vane vacuum pump (2XZ-15C, EVP, Shanghai, China). The iCVD deposition parameters were defined based on prior literature and limited preliminary trials to achieve stable polymerization under steady-state flow conditions and to prevent monomer condensation [27,28]. During deposition, the chamber pressure was maintained at 600 mTorr and monitored by a capacitance manometer (MKS Baratron, Andover, MA, USA), with pressure control achieved via a downstream butterfly valve operated by a PID controller (MKS). Initiator decomposition was achieved using a resistively heated filament array consisting of parallel Ni–Cr filaments (80/20 wt.%, 0.3 mm diameter) positioned approximately 3 cm above the substrates. The filament temperature was maintained at 240 °C and monitored using a K-type thermocouple (Omega, Norwalk, CT, USA). EHA and AA were used as monomers, and tert-butyl peroxide (TBPO) served as the initiator. EHA, AA, and TBPO were vaporized in separate stainless-steel reservoirs held at 70 °C, 40 °C, and room temperature, respectively. The chemical structures of EHA, AA, and TBPO are schematically illustrated in Figure 2b. The precursor vapor flow rates were regulated using needle valves (Swagelok, Solon, OH, USA). The EHA/AA pressure ratio was set to 240/120 for coating the PSA films. The initiator/total monomer ratio (I/M_T_) was kept constant at 1/2. Substrate temperature was maintained at 25 °C using a recirculating water chiller (Lab. Companion RW-0525G, Daejeon, Republic of Korea). Film thickness was monitored in situ by laser interferometry using a 650 nm diode laser and a reference Si wafer. All substrate types investigated in this study were coated simultaneously in a single iCVD deposition run, resulting in PSA thin films with a uniform thickness of approximately 1 µm. All depositions were carried out under identical conditions to ensure uniform film chemistry and thickness across different raster orientations.

### 2.3. Characterization of PSA Thin Films

The chemical structure of the iCVD-deposited PSA thin films was analyzed by Fourier transform infrared spectroscopy (FTIR, Thermo Scientific Nicolet iS20, Waltham, MA, USA) using an ATR accessory. Spectra were collected over the wavenumber range of 4000–400 cm^−1^ with a resolution of 4 cm^−1^ and 32 scans per measurement.

The wettability of the PSA-coated surfaces was evaluated by water contact angle measurements using a contact angle goniometer (Kruss Easy Drop). A 2.0 μL droplet of deionized water was deposited on PSA coated silicon substrate surface, and the static contact angle was recorded at room temperature. Measurements were repeated at multiple locations to ensure reproducibility. The optical transmittance of PSA thin films deposited on glass substrates was measured in the ultraviolet–visible (UV–Vis) spectral range using a UV–Vis spectrophotometer (Shimadzu UV-1800, Shimadzu Inc., Kyoto, Japan), with bare glass used as the reference. The measurements were performed to assess the transparency of the PSA coatings in the visible region and to verify their suitability for optically transparent applications.

For adhesion testing, PSA coatings were applied to only one of the two FDM-printed PLA substrates. The PSA-coated substrate was used as the lower adherend, while an uncoated PLA substrate with identical raster orientation was placed on top and brought into contact at room temperature under gentle manual pressure to ensure intimate interfacial contact. This single-sided coating configuration was intentionally selected to isolate the effect of substrate surface anisotropy on PSA adhesion and to avoid contributions from PSA–PSA interactions. After bonding, the assembled specimens were allowed to equilibrate prior to mechanical testing. The adhesion performance of the PSA-coated PLA substrates was evaluated through standardized mechanical tensile and adhesion tests [26,29]. All tests were conducted under identical environmental conditions to ensure reproducibility, and at least five specimens were tested for each raster orientation to obtain statistically reliable results. Adhesion behavior was assessed under tensile and three-point bending loading modes using single-lap joint configurations. Following mechanical testing, selected specimens were examined by SEM (Zeiss-Gemini 300, Carl Zeiss AG, Jena, Germany) to analyze failure mechanisms and interfacial behavior, with particular emphasis on PSA fibrillation, interfacial debonding, and localized deformation associated with raster orientation. In addition, cross-sectional SEM imaging was employed to confirm the thickness and uniformity of the deposited PSA thin films.

## 3. Results and Discussion

### 3.1. The Film Structure of PSA Thin Film

**Figure 3:**
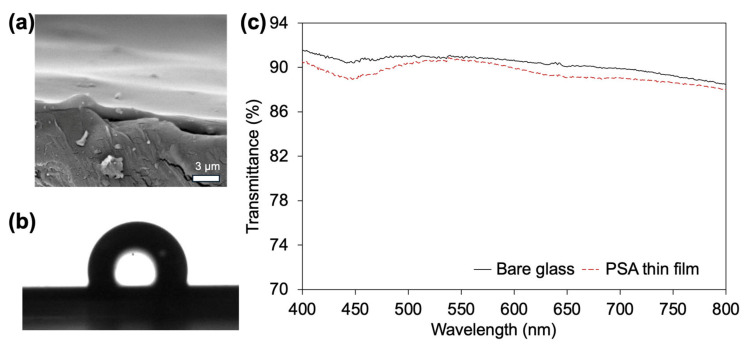
(**a**) Cross-sectional SEM image showing the thickness of the iCVD-deposited PSA thin film, (**b**) water contact angle measurement of the PSA-coated surface, and (**c**) optical transmittance spectra of bare and PSA-coated glass substrates.

The thickness and uniformity of the iCVD-deposited PSA films were examined by cross-sectional SEM. The SEM image (Figure 3a) confirmed that the deposited films exhibited a nearly uniform thickness across the substrate, in good agreement with the nominal values obtained from in situ thickness monitoring during deposition. No noticeable thickness gradients, voids, or delamination were observed, indicating stable deposition conditions and conformal film growth.

The wettability of the PSA-coated Si wafer surface was evaluated by static water contact angle measurements to assess the intrinsic surface wettability of the deposited PSA layer under well-defined conditions. The films exhibited a water contact angle of 106.8°, indicating a moderately hydrophobic surface. This behavior is primarily associated with the dominant EHA segments in the copolymer, while the incorporation of AA introduces polar functional groups that contribute to the surface polarity of the copolymer without dominating its overall hydrophobic character. The coexistence of hydrophobic and polar functionalities reflects the designed copolymer composition and is relevant for understanding the surface characteristics of the PSA thin films.

Optical transmittance measurements were performed to characterize the intrinsic optical quality of the iCVD-deposited PSA thin film, independent of the opaque PLA substrate. The PSA-coated glass exhibited slightly reduced optical transmittance compared to bare glass; however, the films remained highly transparent across the visible region (Figure 3c). The minimal loss in transmittance indicates that the iCVD-deposited PSA layers are optically uniform and free of scattering centers, suggesting their suitability for applications where optical transparency of the adhesive layer is required, such as optical interfaces or transparent bonding layers.

**Figure 4:**
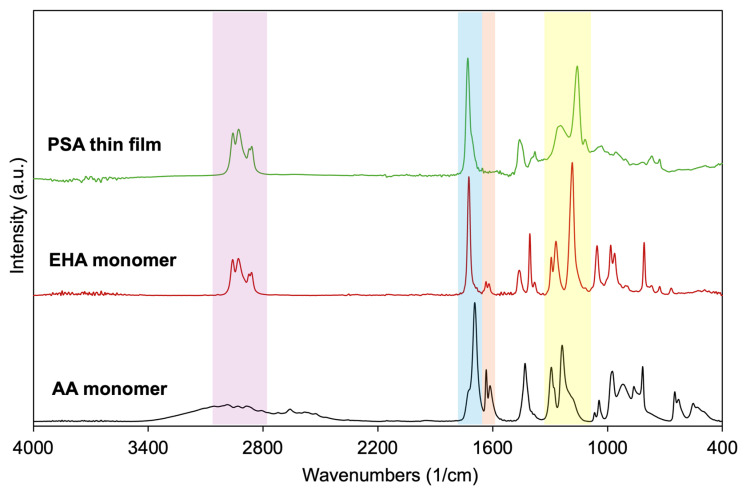
FTIR spectra of EHA, AA monomers, and the iCVD-deposited PSA thin film.

The chemical structure of the iCVD-deposited PSA thin films synthesized from EHA and AA was confirmed by FTIR spectroscopy. The FTIR spectra (Figure 4) exhibited characteristic absorption bands corresponding to both monomer units, indicating successful copolymerization during the iCVD process. The strong carbonyl (C=O) stretching vibration observed in the range of 1650–1750 cm^−1^ is attributed to the ester groups of EHA, while the broad absorption band associated with O–H stretching of carboxylic acid groups confirms the incorporation of AA into the polymer backbone. Additional peaks observed in the 1100–1230 cm^−1^ region, attributed to C–O–C stretching vibrations of ester groups, further support the formation of an acrylate-based PSA network. The absorption bands observed in the 2850–2960 cm^−1^ region are assigned to aliphatic C–H stretching vibrations originating from the EHA side chains and the PSA backbone. Importantly, the absence of characteristic vinyl C=C stretching vibrations, typically observed in the 1550–1650 cm^−1^ region, indicates effective consumption of the double bonds during polymerization, confirming that chain growth occurred through the C=C bonds. These results verify that EHA and AA were successfully integrated into a PSA copolymer structure via iCVD [28,30].

Overall, the iCVD characterization results demonstrate that the EHA–AA-based PSA thin films possess a well-defined chemical structure, smooth surface morphology, high optical transparency, and moderate hydrophobicity. These intrinsic film properties provide a stable and uniform adhesive layer, ensuring that the adhesion performance discussed in the following sections is predominantly governed by the surface morphology and raster orientation of the FDM-printed PLA substrates rather than by variations in the PSA film itself.

### 3.2. Adhesive Performance of PSA Thin Film

**Figure 5:**
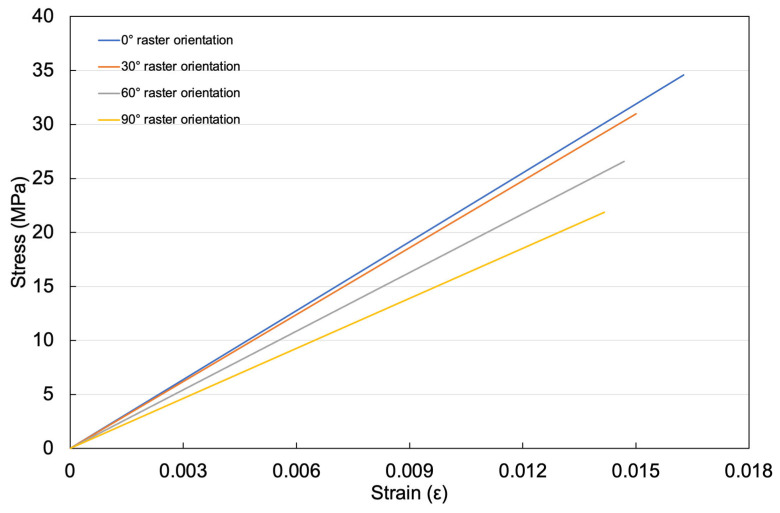
Comparison of the elastic tensile stress–strain response of uncoated FDM-printed PLA substrates with different raster orientations (0°, 30°, 60°, and 90°).

The effect of raster orientation on the mechanical properties of PLA was investigated through tensile testing of uncoated FDM-printed substrates. Representative elastic stress–strain responses for PLA samples fabricated with 0°, 30°, 60°, and 90° raster orientations are shown in Figure 5, while the corresponding elastic modulus and tensile strength values are summarized in Table 1. The initial linear elastic region is shown for clarity and direct comparison of elastic modulus. The results indicate that PLA printed with a 0° raster orientation exhibits the highest elastic modulus (2.14 ± 0.23 GPa) and tensile strength (34.59 ± 3.02 MPa), consistent with load being applied parallel to the filament alignment. With increasing raster orientation, both elastic modulus and tensile strength decrease systematically, reflecting reduced filament continuity in the loading direction. As shown in Figure 5, this orientation-dependent reduction in elastic modulus confirms that substrate compliance varies significantly and must be considered when interpreting the adhesion results presented in this study. Importantly, although tensile strength and adhesion strength exhibit qualitatively similar monotonic trends with raster orientation, their absolute magnitudes differ by more than two orders of magnitude. The tensile strength of uncoated PLA (tens of MPa) is substantially higher than the measured adhesion strength (on the order of 10^−2^ MPa), indicating that failure during adhesion testing is not substrate-limited and that bulk fracture of the PLA substrate does not occur under the applied loading conditions.

**Figure 6:**
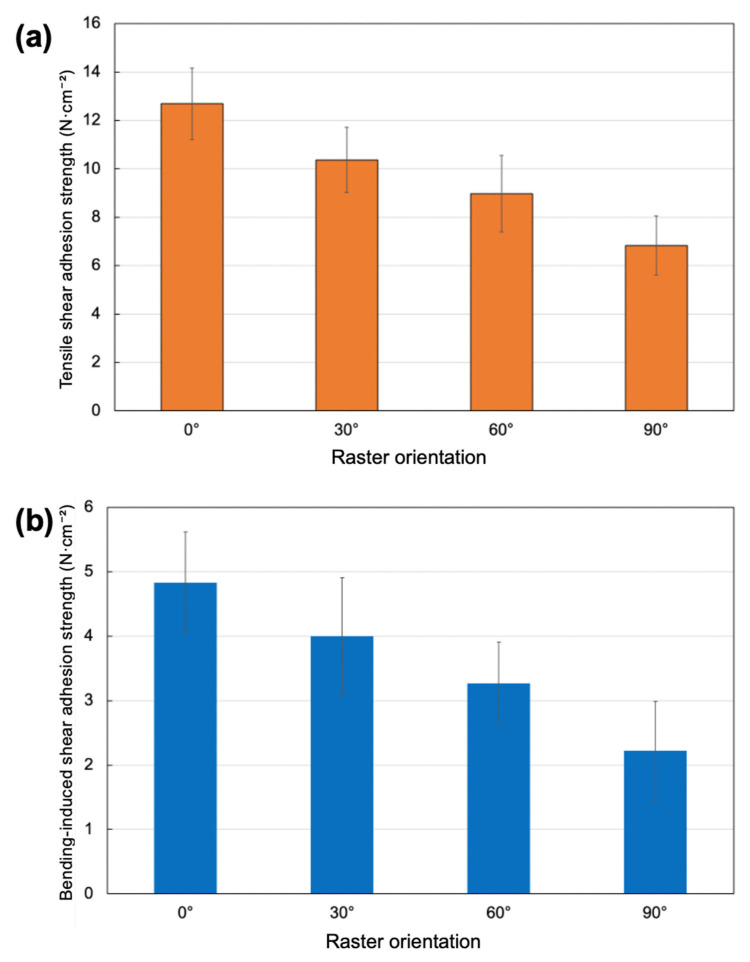
Effect of raster orientation on shear adhesion strength of PSA-coated PLA substrates under (**a**) tensile loading and (**b**) three-point bending.

The adhesion performance of the PSA-coated PLA substrates exhibited a strong dependence on raster orientation under both tensile and three-point bending loading conditions. Raster orientation was the only experimental parameter varied, while all deposition conditions and film thickness were kept constant within experimental reproducibility. In contrast to conventional PSA tests conducted on rigid substrates, the present system involves a compliant polymeric substrate fabricated by FDM. As a result, the measured adhesion response reflects a coupled behavior between PSA viscoelastic deformation and substrate compliance. Therefore, the reported adhesion strength should be interpreted as an apparent adhesion response rather than a purely interfacial strength value. Although substrate deformation contributes to the measured response, raster orientation systematically modifies surface continuity and filament alignment, which governs PSA wetting, fibrillation, and debonding behavior. Consequently, the observed adhesion trends are primarily governed by surface-structure effects rather than by changes in intrinsic PLA mechanical properties. It should be emphasized that this behavior represents a mechanically coupled response, in which substrate compliance influences deformation during testing, while interfacial separation remains the governing failure event rather than substrate fracture. The tensile adhesion results, illustrated in Figure 6, clearly demonstrate that raster alignment relative to the loading direction plays a decisive role in governing shear adhesion strength and deformation behavior.

Under tensile loading, specimens printed with a 0° raster orientation showed the highest maximum force (79.3 N) and shear adhesion strength (12.688 N/cm^2^), accompanied by the largest failure displacement (1.2 mm). This behavior indicates that raster alignment parallel to the loading direction promotes effective interfacial contact and enables extensive viscoelastic deformation of the PSA layer prior to failure [31,32]. The higher displacement at failure further suggests enhanced fibrillation and energy dissipation during debonding. As the raster angle increased, a systematic reduction in tensile adhesion performance was observed. The 30° and 60° specimens exhibited intermediate shear adhesion strengths of 10.368 and 8.976 N/cm^2^, respectively, along with reduced failure displacements. This gradual decline reflects a progressive disruption of surface continuity and anisotropic surface features that limit PSA wetting and fibrillation efficiency. The 90° raster orientation resulted in the lowest tensile adhesion performance, with a maximum force of 42.7 N and a shear adhesion strength of 6.832 N/cm^2^, corresponding to a reduction of approximately 46% compared to the 0° orientation. The lower failure displacement (0.72 mm) indicates premature debonding with limited energy absorption [33].

**Figure 7:**
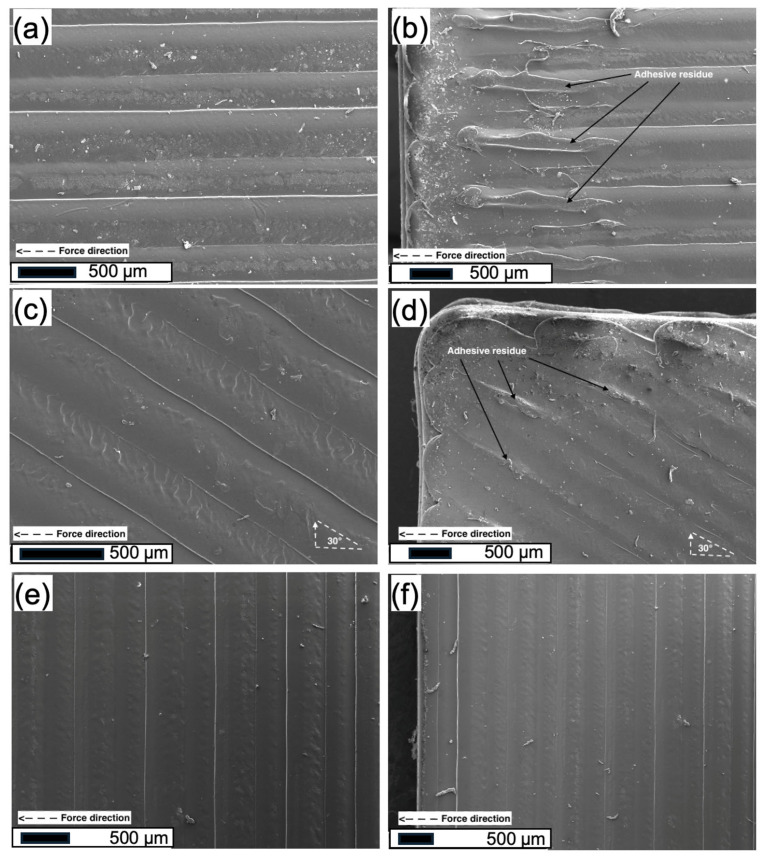
SEM images of PSA-coated FDM-printed PLA substrates after adhesion tests; (**a**,**b**) 0° raster orientation with pronounced adhesive deformation and fibrillation, (**c**,**d**) 30° raster orientation with reduced fibrillation and localized adhesive residues, and (**e**,**f**) 90° raster orientation exhibiting predominantly interfacial separation with minimal adhesive deformation.

SEM analysis (Figure 7) provides microstructural insight into the failure behavior of PSA-coated PLA substrates with different raster orientations. Due to the ultrathin nature of the PSA layer (~1 µm), post-test optical identification of adhesive residue on opposing substrates is not feasible; accordingly, SEM observations are used as qualitative indicators of interfacial deformation and separation behavior rather than definitive failure-mode classification. As shown in Figure 7a, the PSA-coated surface prior to testing preserves the raster-induced surface morphology, indicating that the adhesive layer conforms well to the underlying filament structure without obscuring surface anisotropy. For specimens printed with a 0° raster orientation (Figure 7b), the post-test surfaces exhibit pronounced PSA stretching and fibrillation aligned with the loading direction. The presence of elongated adhesive filaments and distributed adhesive residues on the PLA surface suggests enhanced interfacial interaction and effective stress transfer across the adhesive–substrate interface. This morphology is consistent with extensive viscoelastic deformation of the PSA layer prior to interfacial separation, enabling effective energy dissipation during debonding [34]. At intermediate raster orientations (30°, Figure 7c), PSA fibrillation becomes shorter and more discontinuous. The angled filament arrangement introduces surface discontinuities that locally limit intimate contact between the PSA film and the PLA substrate. As a result, interfacial separation initiates earlier, and the extent of adhesive deformation is reduced compared to the 0° configuration. Nevertheless, the presence of localized adhesive stretching indicates that partial stress redistribution still occurs prior to failure. In contrast, specimens printed with higher raster angles exhibit predominantly interfacial separation with minimal adhesive deformation (Figure 7d). The PSA layer detaches from the PLA surface with limited fibrillation, reflecting insufficient interfacial continuity and reduced effective contact area. This surface morphology correlates with the lower adhesion strength and reduced failure displacement observed for these raster orientations. The interruption of filament continuity perpendicular to the loading direction promotes stress concentration at the interface, leading to premature debonding [33,34,35]. In addition, the SEM images corresponding to the 90° raster orientation (Figure 7e,f) reveal a largely featureless interfacial morphology with minimal observable PSA deformation, consistent with the lowest adhesion strength measured for this orientation. These SEM observations are intended to reflect the overall post-test surface condition rather than localized failure features, in line with the ultrathin nature of the PSA coating.

Overall, SEM observations confirm that raster orientation strongly influences interfacial contact quality and deformation behavior during debonding. Favorable raster alignment promotes extensive adhesive deformation and delayed interfacial separation, whereas unfavorable orientations lead to early separation with limited energy dissipation.

The three-point bending adhesion results exhibited trends consistent with the tensile measurements, further confirming the dominant influence of raster orientation. The highest bending-induced shear adhesion strength was obtained for the 0° specimens (4.832 N/cm^2^), along with the largest failure displacement (11.2 mm). This behavior suggests that aligned raster structures facilitate stable stress redistribution at the adhesive interface under combined bending and shear deformation, allowing the PSA layer to sustain large deformations before failure [36]. With increasing raster angle, both maximum force and shear adhesion strength decreased monotonically. Specimens printed at 30° and 60° showed moderate reductions in adhesion strength and displacement at failure, whereas the 90° specimens exhibited the weakest bending performance, with a shear adhesion strength of 2.224 N/cm^2^ and a failure displacement of 8.1 mm. The reduced deformation capability under bending indicates limited PSA fibrillation and early interfacial debonding associated with surface anisotropy and interrupted filament patterns. As shown in Figure 6b, the adhesion strength decreases monotonically with increasing raster orientation, following a near-linear trend from 0° to 90°. Although the reduction between 0°, 30°, and 60° occurs gradually, these orientations exhibit a consistent decline in adhesion strength, with the lowest values observed for the 90° raster orientation. This trend highlights the cumulative influence of raster-induced surface discontinuities and orientation-dependent substrate compliance on interfacial adhesion performance.

Overall, the combined tensile and bending results demonstrate that raster orientation strongly governs PSA adhesion performance by controlling surface morphology, interfacial contact quality, and deformation mechanisms during debonding [32,36]. Raster orientations aligned with the loading direction enhance shear adhesion strength and failure displacement, whereas perpendicular orientations significantly suppress adhesion efficiency and energy dissipation [37]. Another parameter affected by raster orientation is surface roughness. In the bonding zone, surface structures obtained parallel to the direction of force application reduce surface roughness, thus improving the mechanical properties of the adhesive. In surface structures obtained perpendicular to this direction, the resulting increase in surface roughness weakens the adhesive bonds [38,39,40]. These findings underline the critical importance of raster orientation as a design parameter for FDM-printed substrates intended for PSA-based bonding applications.

## 4. Conclusions

In this study, PSA thin films deposited by iCVD were successfully integrated with FDM-printed PLA substrates to investigate the effect of raster orientation on adhesion performance. The results clearly demonstrate that raster orientation is a dominant factor governing interfacial adhesion behavior. Among the investigated configurations, the 0° raster orientation exhibited the highest adhesion performance, achieving a shear adhesion strength of 12.688 N/cm^2^ under tensile loading and 4.839 N/cm^2^ under three-point bending. In contrast, increasing the raster angle led to a systematic reduction in adhesion strength due to disrupted surface continuity and limited deformation at the adhesive interface. These findings highlight that effective control of raster orientation during FDM printing enables reliable tuning of PSA bonding performance without modifying adhesive chemistry, providing a practical design strategy for adhesively bonded additively manufactured components. This approach is particularly attractive for scalable and solvent-free integration of PSA layers into additively manufactured components.

## Figures and Tables

**Table 1:** Mechanical properties of uncoated FDM-printed PLA substrates with different raster orientations (0°, 30°, 60°, and 90°).

Raster Orientations	Modulus of Elasticity (GPa)	Tensile Strength (MPa)
0°	2.14 ± 0.23	34.59 ± 3.02
30°	2.07 ± 0.19	30.97 ± 3.33
60°	1.94 ± 0.22	26.57 ± 2.94
90°	1.77 ± 0.16	21.87 ± 2.75

## Data Availability

The original contributions presented in this study are included in the article. Further inquiries can be directed to the corresponding author.

## Abbreviations

The following abbreviations are used in this manuscript:

AA	Acrylic acid
AM	Additive manufacturing
EHA	2-ethylhexyl acrylate
iCVD	Initiated chemical vapor deposition
FDM	Fused deposition modeling
PLA	Polylactic acid
PSA	Pressure-sensitive adhesive
